# Exhausting treadmill running causes dephosphorylation of sMLC2 and reduced level of myofilament MLCK2 in slow twitch rat soleus muscle

**DOI:** 10.14814/phy2.12285

**Published:** 2015-02-23

**Authors:** Kristin Halvorsen Hortemo, Jan Magnus Aronsen, Ida G Lunde, Ivar Sjaastad, Per Kristian Lunde, Ole M Sejersted

**Affiliations:** 1 Institute for Experimental Medical Research, Oslo University Hospital and University of OsloOslo, Norway; 2 KG Jebsen Cardiac Research Center and Center for Heart Failure Research, University of OsloOslo, Norway; 3 Bjørknes CollegeOslo, Norway; 4 Department of Genetics, Harvard Medical SchoolBoston, Massachusetts

**Keywords:** Exercise, myosin light chain, protein O-GlcNAcylation, protein phosphorylation

## Abstract

Myosin light chain 2 (MLC2) is a small protein in the myosin complex, regulating muscle contractile function by modulating Ca^2+^ sensitivity of myofilaments. MLC2 can be modified by phosphorylation and O-GlcNAcylation, two reversible and dynamic posttranslational modifications. The slow isoform of MLC2 (sMLC2) is dephosphorylated in soleus muscle during in situ loaded shortening contractions, which correlates with reduction in shortening capacity. Here, we hypothesize that exhausting in vivo treadmill running induces dephosphorylation of MLC2 in slow twitch soleus, but not in fast twitch EDL muscle, and that there are reciprocal changes in MLC2 O-GlcNAcylation. At rest, both phosphorylation and O-GlcNAcylation of MLC2 were lower in slow than fast twitch muscles. One bout of exhausting treadmill running induced dephosphorylation of sMLC2 in soleus, paralleled by reduced levels of the kinase MLCK2 associated to myofilaments, suggesting that the acute reduction in phosphorylation is mediated by dissociation of MLCK2 from myofilaments. O-GlcNAcylation of MLC2 did not change significantly, and seems of limited importance in the regulation of MLC2 phosphorylation during in vivo running. After 6 weeks of treadmill running, the dephosphorylation of sMLC2 persisted in soleus along with reduction in MLCK2 both in myofilament- and total protein fraction. In EDL on the contrary, phosphorylation of MLC2 was not altered after one exercise bout or after 6 weeks of treadmill running. Thus, in contrast to fast twitch muscle, MLC2 dephosphorylation occurs in slow twitch muscle during in vivo exercise and may be linked to reduced myofilament-associated MLCK2 and reduced shortening capacity.

## Introduction

Repeated muscle activity leads to a decline of muscle function known as fatigue. Fatigue typically develops during daily activities like walking or running, and involves decline in muscle force development, shortening and relaxation. However, the mechanisms that mediate fatigue are complex and not fully understood (for review see Allen et al. (2008)). Posttranslational modifications (PTMs) like phosphorylation and O-GlcNAcylation of myofilament proteins can alter protein function and affect fatigue development in working muscles (Fitts 2008; Cieniewski-Bernard et al. 2009). One of these myofilament proteins is regulatory myosin light chain 2 (MLC2), that together with the essential myosin light chain 1 wrap around the neck of the myosin heavy chain, providing mechanical support (Lowey and Trybus 2010).

MLC2 can be modified by phosphorylation at Ser15 by the skeletal muscle myosin light chain kinase (MLCK2) (reviewed by Stull et al. (2011)), and phosphorylation is thought to promote the movement of the myosin head toward actin, increasing the Ca^2+^ sensitivity of the contractile apparatus (Persechini et al. 1985; Stull et al. 2011). We have previously reported dephosphorylation of slow isoform of MLC2 (sMLC2) in slow twitch soleus muscle during in situ loaded shortening contractions (Munkvik et al. 2009; Hortemo et al. 2013). This was well correlated with a decline in muscle shortening (fatigue), suggesting that sMLC2 phosphorylation participate in the regulation of shortening capacity in slow twitch muscle by modulating Ca^2+^ sensitivity of myofilaments. In fast twitch skeletal muscle, tetanic isometric stimulation is associated with increased MLC2 phosphorylation and posttetanic twitch potentiation, while no such potentiation is seen in slow twitch skeletal muscle (Vandenboom et al. 2013). This suggests that MLC2 is differently regulated in fast and slow twitch muscle.

O-GlcNAcylation of skeletal muscle proteins is recently suggested to be a regulator of skeletal muscle function (reviewed by Cieniewski-Bernard et al. *(2014b)*). Several contractile proteins have been described to be O-GlcNAcylated (Cieniewski-Bernard et al. 2004, 2012; Hedou et al. 2007; Ramirez-Correa et al. 2008), including MLC2, and it is believed that phosphorylation and O-GlcNAcylation could interplay (i.e., called phospho-GlcNAc modulation) in tuning the functional properties of MLC2. However, to our knowledge, the effects of exercise on phospho-GlcNAc modulation of MLC2 in skeletal muscle have not been investigated.

Phosphorylation and O-GlcNAcylation are O-linked, reversible and dynamic PTMs at serine and threonine residues, and O-GlcNAcylation is hence different from irreversible N-linked glycosylation in the endoplasmic reticulum-Golgi (for review, see Hart et al. (2011)). The specific site for O-GlcNAcylation on skeletal muscle MLC2 has not been determined, but in rat cardiac MLC2 the O-GlcNAcylation site is the same as the phosphorylation site (Ser15) (Ramirez-Correa et al. 2008), corresponding to the phosphorylation site in rat skeletal muscle MLC2.

The enzymes responsible for phosphorylation–dephosphorylation of MLC2 in skeletal muscle are MLCK2 and myosin light chain phosphatase (MLCP), respectively (reviewed by Stull et al. (2011)). MLCP is composed of the catalytic subunit of protein phosphatase 1 beta (PP1B), the myosin phosphatase targeting protein (MYPT2), and the small unit M20 of unknown function. Modulation of protein O-GlcNAcylation is achieved by two evolutionary conserved enzymes, O-GlcNAc transferase (OGT) and O-GlcNAcase (OGA). OGT and OGA antagonistically add and remove the O-linked GlcNAc to serine or threonine residues, comparative to protein phosphorylation by kinases and phosphatases. Interestingly, MLCK2, MYPT2, PP1, OGT, and OGA were recently shown to exist in a multienzymatic complex at the sarcomere (Cieniewski-Bernard et al. 2014a).

In this study, we hypothesized that the phospho-GlcNAc pattern is different in slow versus fast twitch muscle at rest, and that the effects of in vivo treadmill running on MLC2 phosphorylation are different between the two muscle types. Specifically, an important aim of our study was to determine if there is dephosphorylation of sMLC2 in slow twitch muscle during in vivo running and whether there are reciprocal changes in MLC2 O-GlcNAcylation. Finally, we measured MLCK2, MLCP, OGT, and OGA in the muscle homogenate and in the myofilament protein subfraction since the amount of these enzymes might explain the degree of MLC2 phosphorylation and O-GlcNAcylation.

## Materials and Methods

### Ethical approval

All experiments were performed in accordance with the Norwegian Animal Welfare Act. Protocols were reviewed and approved by the Norwegian Animal Research Authority (ID 3383 and 3301) and conformed to the NIH Guide for the Care and Use of Laboratory Animals. Male Wistar and Sprague Dawley rats (Taconic, Skensved, Denmark) were housed in a controlled environment (temperature 22 ± 2°C, humidity 55 ± 5%, 12/12 h daylight/night cycle) for 1 week after arrival before included in the study. Rats were fed standard rat chow (B & K Universal, Oslo, Norway) and water ad libitum.

### Treadmill running – One exercise bout

Male Wistar rats ∼300 g (*n* = 18) were acclimatized to the treadmill for 15 min the last 2 days prior to the experiment (5 min at 8 m·min^−1^, 10 min at 12 m·min^−1^). At the day of the experiment, rats were randomly assigned to three different groups; the run (RUN) group performed one exercise bout to fatigue on the treadmill; the recovery (REC) group performed one exercise bout and were subsequently allowed 24 h rest, and the control (CTR) group remained sedate. The exercise was performed at 12.5° inclination with incremental running speed, starting at 8 m·min^−1^ and increasing every second min toward maximum running speed of the individual rat. The exercise protocol was continued until exhaustion, defined as when the rat was unable to continue running at the maximum running speed. Rats in the RUN group were at the end of exercise immediately anaesthetized in a chamber with 4% isoflurane (Forene®) and sacrificed by neck dislocation, and within 1 min after termination of the exercise protocol soleus and extensor digitorum longus (EDL) muscles were harvested and snap-frozen in liquid nitrogen and stored at −80°C until analysis. Rats in the REC group were at the end of exercise allowed rest, food, and water ad libitum for 24 h before muscles were harvested.

### In situ exercise protocol

The in situ exercise protocol was performed essentially as described previously (Munkvik et al. 2009). In short, male Wistar rats ∼300 g (*n* = 9) were anaesthetized, intubated and placed on a respirator, and the soleus muscle was prepared in situ keeping the blood supply intact. The distal tendon of soleus was fastened to a combined force and length transducer, and the muscle was intermittently electrically stimulated to perform fatiguing shortening contractions toward a preset load (1/3 of maximal tetanic force). The temperature was kept at 37°C by preheated 0.9% NaCl running over the epimysium of the muscle. At the end of the experiment, the soleus muscle from the stimulated (i.e., exercised; EX) leg was harvested and snap-frozen in liquid nitrogen within 10 sec after termination of contractions, and subsequently the soleus muscle from the resting control (CTR) leg was harvested and snap-frozen within 1 min before the animals were killed by neck dislocation while still anaesthetized.

### Treadmill running – Six weeks

Male Sprague Dawley rats ∼280 g (*n* = 14) were randomly assigned to perform a 6 weeks interval training program (RUN) on the treadmill (Columbus Instruments, Colombus, OH) or to remain sedate (CTR). One week acclimatization was performed with running velocity 6 m**·**min^−1^ for 30, 45, 60, 75, 90, and 120 min, respectively, and 1 day with rest. Interval training was then performed 6 days a week at 25° inclination; 10 min warm-up (10 m·min^−1^) followed by 12 × 8 min intervals separated by 2 min resting periods (6 m·min^−1^). The running speed during intervals was 15 m·min^−1^ the first week, then increasing with 2 m·min^−1^ each week. Rats were given 0.1–0.2 g chocolate (Kvikk Lunsj, Freia, Oslo, Norway) and free access to water after accomplishing each training session. Rats not able to fulfill the exercise protocol were withdrawn from the study. After the last training session (after 6 weeks), rats were allowed rest for 24 h before the muscles were harvested; rats were anesthetized in a chamber and subsequently mask-ventilated by 3% isoflurane and 97% O_2_, and within 3 min after onset of anesthesia the soleus and EDL muscles were dissected and snap-frozen in liquid nitrogen before the animals were sacrificed by cardiac excision while still anaesthetized.

### Protein extraction

Total protein lysates were made by pulverizing muscles in a mortar with liquid nitrogen and subsequently homogenizing with a Polytron® 1200 in ice-cold lysis buffer (20 mmol·L^−1^ Hepes pH 7.5, 150 mmol·L^−1^ NaCl, 1 mmol·L^−1^ EDTA, 0.5% Triton X-100) with protease inhibitors (Complete EDTA-free tablets; Roche Diagnostics, Grenzach, Germany), phosphatase inhibitors (PhosSTOP, Roche Diagnostics), and 40 mmol·L^−1^ glucosamine (Sigma-Aldrich, Oslo, Norway) to provide excess substrate for OGA (Lunde et al. 2012). The homogenates were stored on ice for 30 min before they were centrifuged at 20,000 g for 30 min at 4°C. Supernatants were stored at −80°C until subsequent analysis.

Myofibrillar protein extracts were made by pulverizing muscles in a mortar with liquid nitrogen. Ice cold 6.35 mmol·L^−1^ EDTA solution with protease inhibitors, phosphatase inhibitors, and 40 mmol·L^−1^ glucosamine were added and the muscles samples were homogenized with Polytron® 1200, stored on ice for 30 min, and centrifuged at 18,000 g for 10 min at 4°C. Pellets were washed with 50 mmol·L^−1^ KCl containing protease inhibitors, phosphatase inhibitors, and glucosamine, and centrifuged for another 10 min. The final pellets were resuspended in 50 mmol·L^−1^ KCl containing protease inhibitors, phosphatase inhibitors, and glucosamine, and stored at −80°C.

### Immunoblotting

Protein concentrations in lysates were determined using the Micro BCA Protein Assay Kit (Pierce/Thermo Scientific, Oslo, Norway) and 20–90 *μ*g of protein was loaded on 1.0 mm 4–15% or 15% Tris-HCl gels (Criterion, BIO-RAD, Oslo, Norway). SDS-PAGE and Western blotting were performed essentially as described in the Criterion BIORAD protocol, using PVDF Hybond membranes (Amersham/GE Healthcare, Oslo, Norway). Blots were blocked in either 5% nonfat dry milk or 5% BSA for 1 h at room temperature, and incubated with primary and secondary antibodies overnight at 4°C and 1 h at room temperature, respectively.

Primary antibodies were anti-O-GlcNAc CTD110.6 (MMS-248R; Covance, Oslo, Norway), anti-MLC2 pSer15 (AP08007PU-N; Nordic BioSite, Täby, Sweden), anti-MLC2 (F109.3E1; BioCytex, Oslo, Norway), anti-MLCK2 (sc-9456; Santa Cruz Biotechnology [SCB], Heidelberg, Germany), anti-MYPT2 (sc-292988; SCB), anti-PP1B (ab53315; Abcam, Cambridge, UK), anti-OGT (O6264; Sigma-Aldrich), anti-OGA (SAB4200267; Sigma-Aldrich), anti-GAPDH (sc-20357; SCB), anti-*α*-tubulin (sc-5286; SCB), and anti-CS (ab96600; Abcam). Blots were incubated with appropriate anti-HRP-conjugated secondary antibodies from Southern Biotechnology (Birmingham, AL), developed using the ECL Plus Western Blotting Detection System (Amersham/GE Healthcare) and visualized in the Las-4000 mini (Fujifilm, Stockholm, Sweden). Blots were reprobed after stripping using the Restore Western Blot Stripping Buffer (21059; Thermo Scientific). Quantification of protein band intensity and processing of immunoblots was performed using ImageQuant (GE Healthcare) and Adobe Photoshop CS5.

### Calculation of MLC2 phosphorylation and O-GlcNAcylation

**Figure 1:**
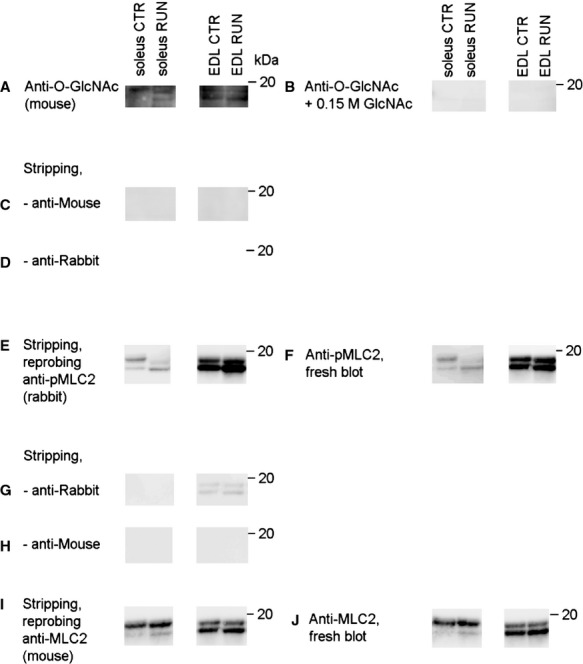
Specificity of antibodies and efficiency of stripping method. Myofilament protein extract from soleus (∼80 *μ*g) and EDL (∼65 *μ*g) muscle from control rat (CTR) and after one exercise bout (RUN) separated on 15% SDS-PAGE, blotted and blocked. (A) Probing with anti-O-GlcNAc antibody (CDT 110.6) and (B) anti-O-GlcNAc antibody together with 0.15 M GlcNAc (GlcNAc, 01140; Sigma-Aldrich) on a parallel blot illustrated the specificity of the anti-O-GlcNAc antibody. The blot in A was stripped with Restore Western Blot Stripping Buffer (Thermo Scientific) for 50 min, washed and blocked, before the blot was reprobed with secondary antibodies (C) anti-Mouse and (D) anti-Rabbit*,* showing efficient stripping of the O-GlcNAc signal. The stripping and blocking procedure was repeated, and (E) the stripped blot and (F) a parallel fresh blot were probed with anti-pMLC2. The same signal pattern was displayed in the reprobed blot as in the fresh blot. The blot in E was then stripped and blocked once more, before probed with secondary antibodies (G) anti-Rabbit and (H) anti-Mouse. Anti-Rabbit produced a weak signal in EDL when the exposure time was increased (G), while anti-Mouse (which was the secondary antibody that should be used in the subsequent probing with anti-MLC2) did not produce any signal. The blot was stripped and blocked one last time, before (I) reprobed with anti-MLC2 in parallel with (J) anti-MLC2 on a fresh blot, showing the same signal pattern in the reprobed blot as in the fresh blot.

**Figure 2:**
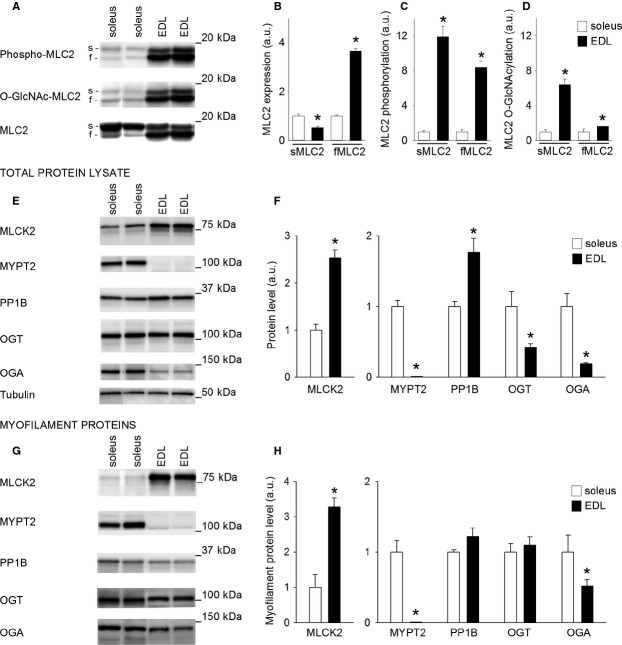
Different phospho-GlcNAc pattern of MLC2 in slow twitch soleus and fast twitch EDL muscle. (A) Representative immunoblots of phosphorylated-, O-GlcNAcylated- and total MLC2 (s, slow isoform; f, fast isoform), and mean data for (B) total- (C) phosphorylated- and (D) O-GlcNAcylated MLC2 in myofilament protein fraction from soleus (white bars) and EDL (black bars). (E) Representative immunoblots of enzymes regulating MLC2 phosphorylation and O-GlcNAcylation analyzed in total protein lysate, and (F) mean data for the enzymes in E. (G) Representative immunoblots of enzymes regulating MLC2 phosphorylation and O-GlcNAcylation analyzed in the myofilament protein fraction, and (H) mean data for the enzymes in G. Data are mean ± SEM. Staining intensities in EDL were calculated relative to the staining intensities in soleus. Tubulin was used as loading control for total protein data, MLC2 for myofilament protein data. *N* = 4 SOL, 4 EDL. *P < 0.05 versus SOL.

O-GlcNAcylation level was detected by a global O-GlcNAc antibody CTD110.6 with subsequent stripping and overlay with MLC2. The phosphorylation level was detected using a site-specific phospho-antibody recognizing MLC2 pSer15. The sequence of probing was; O-GlcNAc, stripping, MLC2 pSer15, stripping, MLC2. The efficiency of stripping and the specificity of antibodies were confirmed in a control experiment (Fig. 1). Further, parallel control blots with anti-O-GlcNAc antibody RL2 (MA1-072; Thermo Scientific) revealed essentially the same pattern as with anti-O-GlcNAc antibody CTD 110.6 (data not shown), but the sensitivity of RL2 to recognize O-GlcNAc-modified proteins was somewhat inferior to CTD 110.6, and we therefore used CTD 110.6 in all our analyses. By using the pan MLC2 antibody (F109.3E1), the different isoforms sMLC2 and fMLC2 could easily be distinguished by their different molecular weight (Fig.2A, lower panel). Staining intensity of sMLC2 and fMLC2 in EDL was calculated relative to the staining intensity of these proteins in soleus to compare the level in fast versus slow twitch muscle, and equal protein loading was ensured by Coomassie staining (not shown).

### Statistics

Data are expressed as means ± SEM relative to control, if not otherwise specified. For all tests, *P* < 0.05 was considered significant. Differences between two groups were tested using Student's paired- or unpaired *t*-test. The statistical analyses were performed by means of SigmaPlot (Systat Software Inc, version 12.5, Erkrath, Germany) or Microsoft Excel 2010 (Microsoft, Oslo, Norway).

## Results

### Different phospho-GlcNAc pattern of MLC2 in soleus versus EDL

MLC2 isoform distribution, MLC2 phosphorylation, and MLC2 O-GlcNAcylation were measured by immunoblotting in soleus and EDL (Fig.2A). As expected, the expression of sMLC2 was highest in soleus, while fMLC2 was most abundant in EDL (Fig.2B). Interestingly, in resting muscle both sMLC2 and fMLC2 phosphorylation (Fig.2C) and O-GlcNAcylation (Fig.2D) were significantly higher in fast twitch EDL compared to slow twitch soleus.

To assess levels of enzymes regulating MLC2 phosphorylation and O-GlcNAcylation, MLCK2 (90 kDa), MYPT2 (110 kDa), PP1B (36 kDa), OGT (110 kDa), and OGA (130 kDa) in total protein lysate from resting soleus and EDL muscles were measured by immunoblotting (Fig.2E). The level of MLCK2 was more than two times higher in EDL compared to soleus (Fig.2F), while MYPT2 barely was detectable in EDL, but abundant in soleus. Further, the expression of both OGT and OGA were significantly lower in EDL compared to soleus.

**Figure 3:**
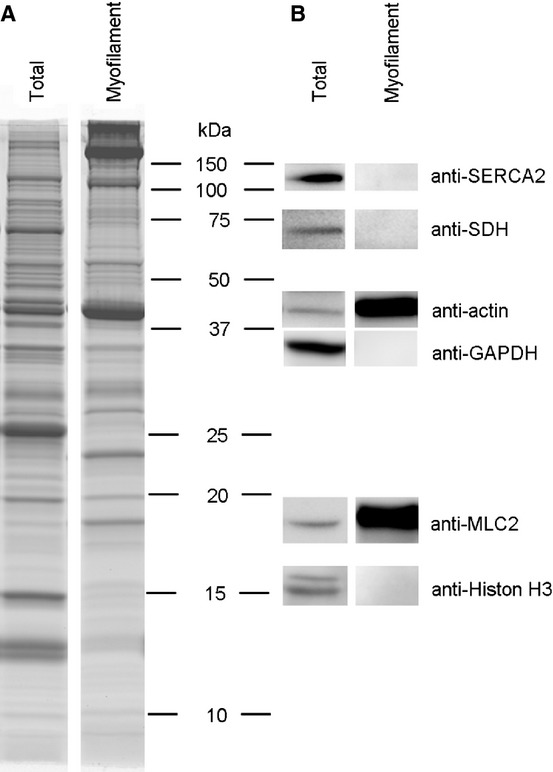
Protein content of total protein lysate and myofilament protein extract from rat soleus muscle. (A) Total protein lysate (Total) and myofilament protein extract (Myofilament) from control rat soleus muscle separated on 15% SDS-PAGE and the protein pattern revealed by Sypro Ruby staining. (B) Total protein lysate (Total) and myofilament protein extract (Myofilament) analyzed by western blot to identify protein markers of different subcellular fractions. Sarco/endoplasmic reticulum Ca^2+^-ATPase (SERCA2) was used as a marker of membrane proteins, succinate dehydrogenase (SDH) as a mitochondrial protein marker, actin and MLC2 as markers of myofilament proteins, GAPDH as a cytosolic marker and Histone H3 as a nuclear protein marker. The blots show that the myofilament fraction contained high levels of myofilament proteins (actin and MLC2), while no SERCA2, SDH, GAPDH or Histone H3 were detected.

In the myofilament protein fraction from soleus and EDL (Fig.2G) all the enzymes analyzed in the total protein extract were detected, suggesting that each enzyme can be found in conjunction with the contractile apparatus. The level of MLCK2 in the myofilament fraction (i.e. myofilament MLCK2) was more than three times higher in EDL than in soleus, and MYPT2 was abundant in soleus but barely detectable in EDL (Fig.2H), well compatible with the higher MLC2 phosphorylation in EDL compared to soleus (Fig.2C). Further, the level of OGA on myofilaments was lower in EDL compared to soleus, which fits the higher O-GlcNAc level of MLC2 in EDL (Fig.2D). Successful fractioning of myofilament proteins was confirmed by Sypro Ruby gel staining and immunoblotting with marker proteins of different subcellular compartments (Fig. 3).

### One bout of treadmill running causes dephosphorylation of sMLC2 in soleus

**Figure 4:**
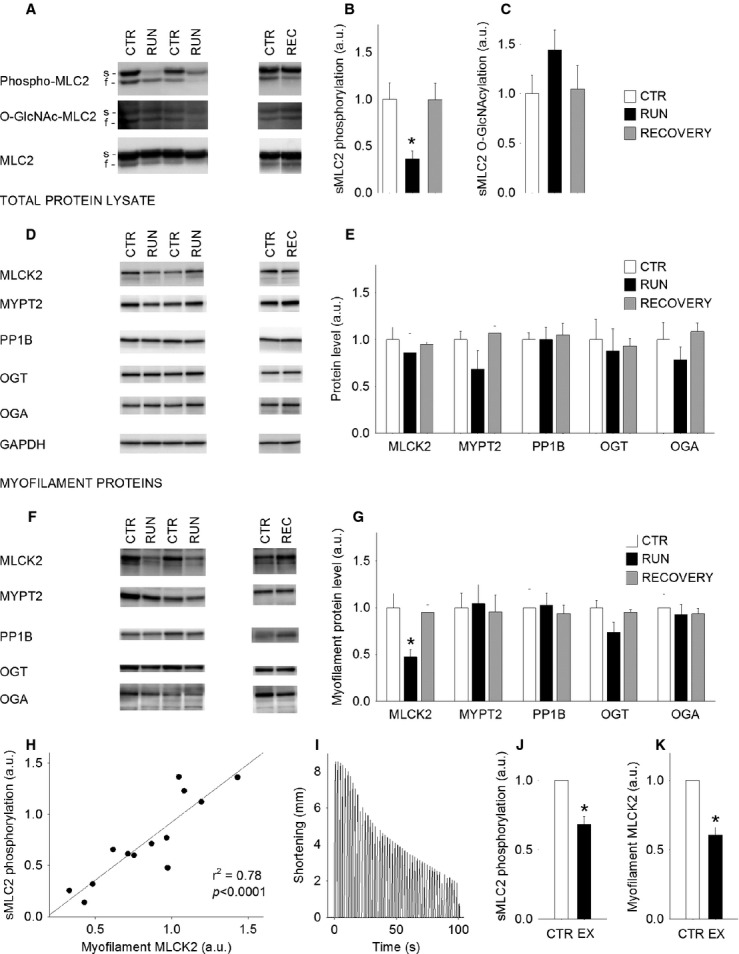
One bout of treadmill running causes dephosphorylation of sMLC2 in soleus that is correlated with reduced myofilament MLCK2. (A) Representative immunoblots, and average data of (B) phosphorylated and (C) O-GlcNAcylated sMLC2 in soleus resting control (white bars, CTR), after one exercise bout (black bars, RUN) and after 24 h recovery (gray bars, REC). (D) Representative immunoblots of enzymes regulating MLC2 phosphorylation and O-GlcNAcylation, analyzed in total protein lysate, and (E) mean data for the enzymes in D. (F) Representative immunoblots of enzymes regulating MLC2 phosphorylation and O-GlcNAcylation, analyzed in the myofilament protein fraction, and (G) mean data for the enzymes in F. (H) The variation in sMLC2 phosphorylation correlated strongly to the level of MLCK2 in the myofilament fraction. (I) Shortening tracing of a representative 100 sec in situ exercise protocol, (J) sMLC2 phosphorylation and (K) myofilament MLCK2 in resting control soleus muscle (white bars, CTR) compared to after 100 sec in situ exercise (black bars, EX). GAPDH was used as loading control for total protein data, MLC2 for myofilament protein data. Data are mean ± SEM relative to CTR. *N* = 5 (CTR), 6 (RUN), 4 (REC), 5 (100 sec, MLC2 phosphorylation), 8 (100 sec, MLCK2). *P < 0.05 versus CTR. s, slow isoform; f, fast isoform.

Maximum running speed of the animals that performed one exhausting exercise bout (*n* = 10) was on average 20 ± 1 m·min^−1^, and the time to exhaustion was 26 ± 1 min. In soleus, phosphorylation of sMLC2 was significantly decreased after one exhausting bout of treadmill running (RUN), and was restored to control values after 24 h recovery (REC) (Fig.4A and B). O-GlcNAcylation of sMLC2 was nominally, but not significantly increased after one exercise bout (*P* = 0.07), and was not different from control after 24 h recovery (Fig.4C).

### sMLC2 phosphorylation is strongly correlated to myofilament MLCK2

One short exercise bout is not expected to alter total protein expression, and accordingly the enzyme expression in the total protein lysate did not change after one exhausting exercise bout (Fig.4D and E). However, in the myofilament fraction, the level of MLCK2 was significantly reduced after one exercise bout (RUN), and was restored to control level after 24 h recovery (REC) (Fig.4F and G). There were no concomitant changes in MYPT2, PP1B, OGT, or OGA. Thus, the variation in sMLC2 phosphorylation after exercise and recovery covaried with the presence of MLCK2 associated to myofilaments, showed by a positive correlation (*P* < 0.0001) (Fig.4H).

### The reduction in myofilament MLCK2 is rapid

To further investigate the association between sMLC2 phosphorylation and myofilament MLCK2, we included experiments using the in situ exercise protocol. Already after 100 sec in situ exercise (EX), there was parallel reduction in muscle shortening (Fig.4I) and sMLC2 phosphorylation (Fig.4J), and in accordance with the results from the in vivo treadmill exercise, the reduction in sMLC2 phosphorylation was accompanied by reduced levels of myofilament MLCK2 (Fig.4K). Thus, reduction in myofilament MLCK2 is linked to reduced sMLC2 phosphorylation also in the in situ model, indicating a rapidly responding mechanism in shortening muscle that performs work.

### Six weeks treadmill running induces persistent dephosphorylation of sMLC2 in soleus

Rats that accomplished the 6 week interval training significantly increased their running speed during intervals from 15 m·min^−1^ to 25 m·min^−1^, and had lower body weight compared to sedate controls (339 ± 9, *n* = 6 vs. 444 ± 9 g, *n* = 6; *P* < 0.05). Expression of CS, a mitochondrial enzyme catalyzing the first reaction in the citric acid cycle and a marker of muscle oxidative capacity, was increased in soleus by 63 ± 4% (*n* = 4 + 4; *P* < 0.001) in the exercise group compared to sedate controls, indicating increased oxidative capacity of the trained muscles.

**Figure 5:**
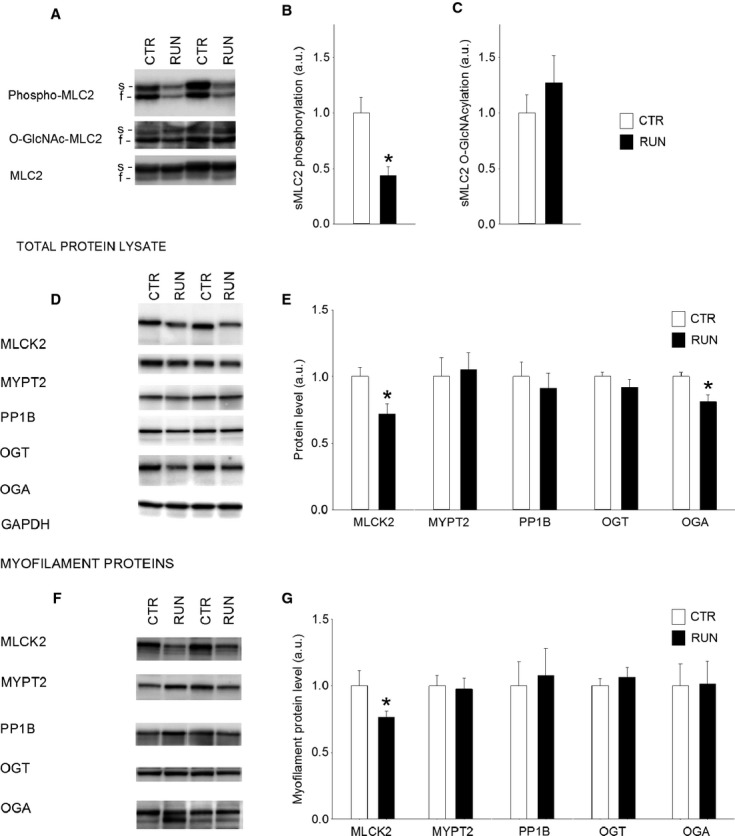
Six weeks treadmill running induces persistent dephosphorylation of sMLC2 in soleus. (A) Representative immunoblots, and average data of (B) phosphorylated and (C) O-GlcNAcylated sMLC2 in soleus resting control (white bars, CTR) and after 6 weeks exercise training (black bars, RUN). (D) Representative immunoblots of enzymes regulating MLC2 phosphorylation and O-GlcNAcylation, analyzed in total protein lysate, and (E) mean data for the enzymes in D. (F) Representative immunoblots of enzymes regulating MLC2 phosphorylation and O-GlcNAcylation, analyzed in the myofilament fraction, and (G) mean data for the enzymes in F. GAPDH was used as loading control for total protein data, MLC2 for myofilament protein data. Data are mean ± SEM relative to control. *N* = 6 (CTR), 6 (RUN).*P < 0.05 versus CTR. s, slow isoform; f, fast isoform.

Muscles were harvested 24 h after the last day of the 6 weeks exercise program. Hence, acute effects of exercise were not investigated in this cohort, but the focus was on long term effects of exercise training. In soleus, sMLC2 phosphorylation was significantly and persistently decreased after 6 weeks exercise training (RUN) compared to sedate controls (CTR) (Fig.  5A and B). The sMLC2 O-GlcNAcylation was not altered in RUN compared to CTR (Fig.5C).

The persistently reduced sMLC2 phosphorylation after 6 weeks exercise training was paralleled by sustained reduction of MLCK2, both in the total protein lysate (Fig.5D and E) and in the myofilament fraction (Fig.5F and G). OGA was reduced in the total protein lysate (Fig.5D and E). The reduced expression of MLCK2 and OGA in the total protein lysate indicates enzyme regulation at the transcriptional level after 6 weeks exercise training, different from after one exercise bout.

### Treadmill running does not affect phospho-GlcNAc pattern of MLC2 in fast twitch EDL

**Figure 6:**
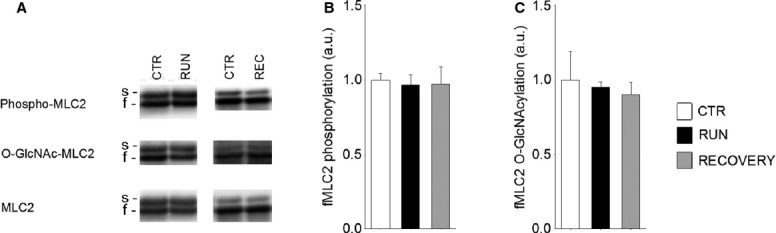
In fast twitch EDL muscle, treadmill running does not affect the phospho-GlcNAc pattern of MLC2. (A) Representative immunoblots of phosphorylated- and O-GlcNAcylated MLC2 in EDL and average data of (B) phosphorylated- and (C) O-GlcNAcylated fMLC2 in resting control (white bars, CTR), after one exercise bout (black bars, RUN) and after 24 h recovery (gray bars, REC). Data are mean ± SEM relative to CTR. MLC2 was used as loading control. *N* = 4 (CTR), 5 (RUN), 4 (REC). s, slow isoform; f, fast isoform.

Remarkably, in contrast to soleus, no differences were found in fMLC2 phosphorylation (Fig.6A and B) or fMLC2 O-GlcNAcylation (Fig.6C) in EDL after one bout of treadmill running or after recovery. In accordance with this, none of the enzymes analyzed (MLCK2, MYPT2, PP1B, OGT, OGA) were altered after one exercise bout in EDL, neither in the total protein lysate nor in the myofilament fraction (data not shown). There was neither any significant difference in phospho-GlcNAc pattern of MLC2 or enzyme expression in EDL after 6 weeks exercise training (data not shown).

## Discussion

In this study we show dephosphorylation of the regulatory protein sMLC2 in slow twitch soleus muscle after exhausting in vivo treadmill running. The phosphorylation level was strongly correlated to the level of the kinase MLCK2 associated to myofilaments, indicating a rapid mechanism to regulate contractile function. O-GlcNAcylation of MLC2 did not change significantly, and seems less important in the regulation of MLC2 phosphorylation during in vivo exercise. The pattern of MLC2 phosphorylation in slow twitch muscle is different from the pattern in fast twitch muscle, and our data support that dephosphorylation of sMLC2 in slow twitch muscle may be linked to reduced shortening capacity of the muscle.

### Basal phospho-GlcNAc pattern is significantly different in soleus vs. EDL

The comparison between soleus and EDL revealed manyfold higher levels of MLC2 phosphorylation and O-GlcNAcylation in EDL compared to soleus, corresponding with profound differences in the expression of regulating enzymes. This suggests that the functional role of phosphorylation and O-GlcNAcylation may be different in the two muscle types. In EDL, the high expression of MLCK2 and the barely detectable level of MYPT2 favor a high phosphorylation level of MLC2. In soleus, on the contrary, the low levels of MLCK2 combined with abundant expression of MYPT provide a plausible explanation of the lower phosphorylation level of MLC2 in soleus. Consistent differences in enzyme activity (Moore and Stull 1984) and expression (Ryder et al. 2007) of MLCK2 and MLCP/MYPT2 between EDL and soleus have been reported previously, supporting a fiber type-specific regulation of MLC2 phosphorylation. Also in regard to O-GlcNAcylation we find differences in enzyme levels, especially lower OGA in the myofilament fraction in EDL compared to soleus, well compatible with the higher O-GlcNAcylation level of MLC2 in EDL.

The higher level of both phosphorylation- and O-GlcNAcylation of MLC2 in fast compared to slow twitch muscle is interesting in light of a recent study which suggest that no phosphorylated form of sMLC2 is at the same time O-GlcNAcylated and vice versa (Cieniewski-Bernard et al. 2014a). If the two PTMs are mutually exclusive, our results suggest that the proportion of unmodified MLC2 is high in soleus, while on the contrary a large part of the total MLC2 in EDL is modified by either phosphorylation or O-GlcNAcylation. The phosphorylation site on skeletal muscle MLC2 is Ser15, while the specific site for O-GlcNAcylation has not been identified. However, in cardiac muscle the only known O-GlcNAcylation site is the same as the phosphorylation site (Ser15) (Ramirez-Correa et al. 2008), and it remains to determine whether this is the case also for skeletal muscle MLC2.

### Reversible dephosphorylation of sMLC2 in soleus after one bout of treadmill running

An important finding of the present study was the reduced phosphorylation of sMLC2 in slow twitch soleus muscle after one bout of in vivo exhausting treadmill running, fully reversible after 24 h rest. This finding supports the results from our previous in situ studies (Munkvik et al. 2009; Hortemo et al. 2013). In these studies, repetitive in situ loaded shortening contractions of soleus induced reversible reduction in muscle shortening correlated with reversible dephosphorylation of sMLC2, suggesting a role of sMLC2 in regulating shortening contractions in slow twitch muscle. Interestingly, a role of MLC2 in regulating loaded shortening contractions has also been reported in cardiac muscle (Sanbe et al. 1999; Scruggs and Solaro 2011; Toepfer et al. 2013).

Most previous experiments conducted on slow twitch muscle in regard to sMLC2 phosphorylation have comprised in vitro or in situ isometric stimulation of short duration, in unfatigued muscle. We have recently shown that loaded shortening contractions (concentric contractions, i.e. work) that were associated to high metabolic stress (drastic fall in muscle CrP, ATP and increase in lactate) seem necessary to induce changes in sMLC2 phosphorylation in slow twitch muscle (Hortemo et al. 2013). There was on the contrary no or little alteration of sMLC2 phosphorylation in slow twitch muscle when the muscle performed solely isometric contractions or when shortening was almost unloaded (Danieli-Betto et al. 2000; Hortemo et al. 2013), where in both situations the metabolic stress is low. This implies that shortening contractions trigger dephosphorylation of MLC2 in slow twitch muscle only when the muscle performs work that causes a metabolic stress, like the exhausting treadmill running performed in the present study.

We demonstrate in the present study that the variation in sMLC2 phosphorylation after one in vivo exercise bout correlates strongly to the level of myofilament associated MLCK2 (Fig.4H). The observation is strengthened by the additional in situ experiments where we find parallel reductions in muscle shortening, sMLC2 phosphorylation and myofilament MLCK2 already after 100 sec (Fig.4I–K). This reveals a rapidly responding system, and we suggest that the reduction in myofilament MLCK2 represents dissociation of the enzyme from the myofilaments, reducing the amount of available kinase in the proximity of sMLC2. MLCK2 was recently shown to exist in a multienzymatic complex at the sarcomere together with MLC2, OGT, OGA, MYPT2, and PP1 (Cieniewski-Bernard et al. 2014a), supporting our finding of MLCK2 in the myofilament protein fraction. However, the specific binding site for MLCK2 on myofilaments remains to be identified.

Also the regulation of MLCK2 in slow twitch skeletal muscle is poorly understood. In fast twitch muscle, the same Ca^2+^ signal that initiates force development also regulates MLCK2 activity; when calmodulin is saturated with four Ca^2+^, the Ca^2+^/calmodulin complex bind to MLCK2 and the regulatory segment on MLCK2 is displaced, allowing interaction with MLC2 (Gao et al. 1995; Ryder et al. 2007; Stull et al. 2011). Exercise could elevate Ca^2+^/calmodulin and activate MLCK2, but this does not fit the reduced phosphorylation of MLC2 that we observed in slow twitch soleus muscle after in situ stimulation (Munkvik et al. 2009; Hortemo et al. 2013) and after in vivo exercise in the present study. In cardiac muscle, there are ambiguous results whether the cardiac MLCK2 is Ca^2+^/calmodulin-dependent or not (reviewed by Scruggs and Solaro (2011)). Our data suggest that Ca^2+^ activation of MLCK2 does not seem to be a major regulator of the activity-dependent phosphorylation level of sMLC2 in slow twitch muscle.

Dynamic interplay between phosphorylation and O-GlcNAcylation of MLC2 has been suggested to participate in the regulation of skeletal and cardiac muscle contractile function (Hedou et al. 2007; Ramirez-Correa et al. 2008; Cieniewski-Bernard et al. 2009, 2012, 2014a; Lunde et al. 2012). In our model of in vivo treadmill running, we did not detect significant changes in O-GlcNAcylation of MLC2. Thus, MLC2 O-GlcNAcylation does not seem to be an important regulator of MLC2 phosphorylation during treadmill running although there was a trend toward increased MLC2 O-GlcNAcylation after one bout of treadmill running (*P* = 0.07). We cannot exclude that this is a type II error since the stoichiometry of O-GlcNAcylation is low and the immunoblot signal of O-GlcNAcylated MLC2 in soleus is weak. Hindlimb unloading was recently reported to induce a 400% increase in sMLC2 phosphorylation in soleus, but only a 50% reduction in O-GlcNAcylation (Cieniewski-Bernard et al. 2014a), suggesting that variations in MLC2 O-GlcNAcylation are smaller than variations in phosphorylation. More sensitive analysis methods and development of site-specific anti-O-GlcNAc-antibodies are warranted to detect subtle variations in protein O-GlcNAcylation.

### Persistent dephosphorylation of sMLC2 in soleus after six weeks treadmill running

In contrast to the full recovery of sMLC2 phosphorylation observed in soleus muscle 24 h after one single exercise bout on the treadmill, sMLC2 was still dephosphorylated 24 h after the last training session following 6 weeks treadmill running. Also different from after one single exercise bout, there was reduction in MLCK2 not only on myofilaments, but also in total protein homogenate, indicating regulation at the transcriptional level after 6 weeks treadmill running and providing a plausible explanation to the persistent dephosphorylation of MLC2.

The animals increased their running speed significantly after 6 weeks exercise, and the increased expression of CS confirmed the training response biochemically. The persistent dephosphorylation of sMLC2 could hence be a component of an advantageous physiological adaption to exercise. We speculate that the persistent dephosphorylation provides a beneficial restraint during long-lasting exercise; postponing the fatigue development by limiting the initial work performed and hence energy consumption. Abbate et al. (2001) showed that the contraction economy (muscle force/muscle energetics cost) was reduced when MLC2 was phosphorylated compared to nonphosphorylated in fast twitch muscles from wild-type and MLCK2 knockout mice. This may suggest that during prolonged activity, increased phosphorylation could cause adverse metabolic changes, and that low levels of MLC2 phosphorylation in slow twitch soleus contribute to the fatigue resistance in this muscle type.

### Phospho-GlcNAc pattern of fast twitch EDL is not altered by treadmill running

In fast twitch EDL muscle, in contrast to slow twitch soleus, the phospho-GlcNAc level of MLC2 was not modulated by treadmill running, and there was no change in enzyme expression. This strongly indicates that the regulation of phospho-GlcNAc of MLC2 in fast twitch EDL is different from the regulation in slow twitch soleus. The profound differences in enzyme levels between soleus and EDL (Fig.2H) could possibly by large explain the dissimilar modulation of MLC2 during exercise, and are likely important for the muscles’ functional properties.

Our results highlight the importance of exploring slow twitch muscle, not only fast twitch muscle as many investigators do, because the response to exercise and the mechanisms of fatigue appear to be fundamentally different in the two muscles. Moreover, to study loaded shortening (concentric) contractions at physiological temperature (37°C) is essential to understand the fatigue development observed during daily life activities.

## Conclusion

In conclusion, we report dephosphorylation of sMLC2 in rat slow twitch muscle after exhausting in vivo treadmill running, both in a single exercise bout and after 6 weeks training. The reduction in sMLC2 phosphorylation is strongly correlated to reduced level of myofilament MLCK2, suggesting a novel mechanism to regulate contractile function. O-GlcNAcylation of MLC2 did not change significantly and seems of less importance in regulating MLC2 phosphorylation during treadmill running. In fast twitch EDL, the levels of phosphorylation and O-GlcNAcylation of MLC2 are higher than in slow twitch soleus at rest, but were not altered by treadmill running. Thus, in contrast to fast twitch muscle, sMLC2 dephosphorylation occurs in slow twitch muscle during in vivo exercise and may be linked to reduced level of myofilament associated MLCK2 and reduced shortening capacity. This provides an exciting basis for discovery of mechanisms underlying fatigue in vivo during loaded shortening contractions like walking and running.
